# Analysis of Subclinical Thyroid Dysfunction and Metabolic Abnormality in 28568 Healthy People

**DOI:** 10.1155/2023/5216945

**Published:** 2023-10-16

**Authors:** Yan Xie, Zhixue Wang, Zongtao Chen

**Affiliations:** ^1^Health Management Centre, The First Affiliated Hospital of Army Medical University, Chongqing 400038, China; ^2^Department of Clinical Laboratory, Bishan Hospital of Chongqing Medical University (Bishan Hospital of Chongqing), Chongqing 402760, China

## Abstract

We analyzed the detection rates of metabolic syndrome (MetS) and subclinical thyroid dysfunction, including subclinical hyperthyroidism (SCHyper) and subclinical hypothyroidism (SCH), in healthy people, as well as their relationship. Clinical data were collected from 28,568 healthy individuals who underwent physical examinations. The detection rates of SCHyper, SCH, and MetS, as well as in different genders and ages, were analyzed. The detection rate of SCHyper and SCH in females was significantly higher than that in males (*P* < 0.001), but that of MetS in males was significantly higher than that in females (*P* < 0.001). In each age group, the detection rate of SCH in females was higher than that in males (*P* < 0.001). The detection rate of SCH was significantly different in different age groups (*P* < 0.001). The detection rates of hyperlipidemia (*P* < 0.001), obesity (*P* = 0.004), hypertension (*P* = 0.009), and hyperglycemia (*P* < 0.001) in the female SCH group were significantly higher than those in the normal group. The detection rates of hyperlipidemia (*P* = 0.006), obesity (*P* = 0.04), and hypertension (*P* = 0.04) in the male SCH group were higher than those in the normal group. The males with SCHyper were more prone to hyperlipidemia (*P* = 0.02) and obesity (*P* = 0.03). In addition, the female SCHyper group was not significantly different from the normal group (*P* > 0.05). Conclusively, the detection rate of SCHyper and SCH in females is higher than that in males, which increases with age. Attention should be paid to subclinical thyroid dysfunction in elderly people, especially females. Early individualized screening and early intervention should be carried out for people with abnormal metabolism.

## 1. Introduction

The thyroid, being the largest endocrine organ in the human body, plays a crucial role in regulating metabolism through the secretion of thyroid hormones [1]. Subclinical thyroid dysfunction, an insidious disease that lacks apparent clinical symptoms, is often detected during a physical examination and results from imbalances in thyroid hormone levels [2]. Clinically, subclinical thyroid dysfunction is divided into subclinical hypothyroidism (SCH) and subclinical hyperthyroidism (SCHyper) [3, 4]. Subclinical thyroid dysfunction is common in females, the elderly, and in areas of iodine deficiency or excess [5, 6].

SCH is a biochemical diagnosis characterized by an elevated serum level of thyroid-stimulating hormone (TSH), while free thyroxine (FT4) levels remain within the normal range [7]. Thyroid hormones exert their effects on the heart through various mechanisms; therefore, SCH has been linked to several risk factors for cardiovascular disease, including hypertension and dyslipidemia [8]. Consequently, even slight fluctuations in hormone levels have the potential to adversely affect both the cardiovascular and metabolic systems [9]. Furthermore, SCH has been found to be associated with insulin resistance and metabolic syndrome (MetS) [10]. Conversely, SCHyper is defined by a concentration of TSH below the normal range, while FT4 concentrations remain within the normal range [11]. Cross-sectional and longitudinal population-based studies have demonstrated that SCHyper is associated with an increased risk of atrial fibrillation, osteoporosis, as well as cardiovascular and all-cause mortality [12, 13].

Numerous studies have examined the association between subclinical thyroid dysfunction and MetS [14–16]. However, these studies provide conflicting findings, likely due to insufficient statistical power. Moreover, there is a dearth of data on the prevalence of subclinical thyroid dysfunction and metabolic abnormalities in the healthy population. Therefore, this study aims to thoroughly analyze the detection rate of subclinical thyroid dysfunction and metabolic abnormalities in a large cohort of healthy individuals. Our findings may provide a basis for the detection of subclinical thyroid dysfunction and for the prevention and treatment of metabolic abnormalities in healthy populations.

## 2. Materials and Methods

### 2.1. Study Participants

This retrospective study enrolled healthy individuals who underwent physical examinations in the Health Management Centre of the First Affiliated Hospital of Army Medical University and the Department of Clinical Laboratory of Bishan Hospital of Chongqing Medical University from January 2018 to January 2022. Inclusion criteria are as follows: (1) subjects aged 18–91 years and (2) subjects had complete and reliable clinical data, including those of thyroid function, body mass index (BMI), blood pressure, blood glucose, blood lipids, and kidney function. Exclusion criteria are as follows: (1) subjects with a confirmed diagnosis of hyperthyroidism or hypothyroidism, or who had received treatment for the above diseases; (2) subjects with severe heart/liver/kidney insufficiency, severe anemia, or malignant tumor; (3) subjects who had no obvious symptoms and whose physical examination results were at the critical value but the results returned to normal after 2-3 months of follow-up; and (4) subjects who received drug therapy that may affect the detection of thyroid function, such as propranolol, metoclopramide, and glucocorticoids. This study was approved by the Ethics Committee of the First Affiliated Hospital of Army Medical University (No.: (B) KY2022188). Informed consent was waived due to the retrospective nature of this study.

### 2.2. Data Collection

The basic data such as age, gender, height, weight, diastolic blood pressure (DBP), and systolic blood pressure (SBP) were collected, as well as blood biochemical results.

### 2.3. Detection of Blood Biochemical Indicators

Venous blood was collected from each subject after fasting for 10 h–14 h and then subjected to detection of blood biochemical indicators. The serum levels of free triiodothyronine (FT3), triiodothyronine (T3), thyroid peroxidase antibody (TPO-Ab), thyroglobulin protein antibody (TG-Ab), thyroid microsomal antibody (Tm-Ab), FT4, total thyroxine (T4), and TSH were detected using an electrochemiluminescence immunoassay system (Cobas E602, Roche). The normal reference ranges were: TSH 0.27∼4.20 mIU/m, FT3 3.1∼6.8 pmol/L, and FT4 12∼22 pmol/L. The serum levels of fasting blood glucose (FBG), high-density lipoprotein cholesterol (HDL-C), low-density lipoprotein cholesterol (LDL-C), triglyceride (TG), total cholesterol (TC), blood urea nitrogen (BUN), creatinine (CR), and uric acid (UA) were measured with an automatic biochemical analyzer (Cobas c701, Roche).

### 2.4. Diagnostic Criteria

According to the recommendations on MetS formulated by the Metabolic Syndrome Research Collaborative Group of the Diabetes Society of the Chinese Medical Association in 2016 [17], MetS can be diagnosed if any 3 or more of the following 5 items are met: (1) central obesity and/or abdominal obesity: waist circumference for men ≥ 90 cm and women ≥85 cm. (2) Hyperglycemia: FBG ≥ 6.1 mmol/L (110 mg/dL) or two-hour postprandial blood glucose ≥ 7.8 mmol/L (140 mg/dL) or subjects were under treatment for diabetes. (3) Hypertension: blood pressure ≥ 130/85 mm Hg and/or subjects were under treatment for hypertension. (4) Fasting TG ≥ 1.70 mmol/L (150 mg/dL). (5) Fasting HDL-C < 1.0 mmol/L (40 mg/dL). If TSH is ≥ 4.2 mIU/ml but FT4 is within the normal range, SCH could be diagnosed. If TSH ≤ 0.27 mIU/ml but FT4 are within the normal range, SCHyper could be diagnosed.

### 2.5. Statistical Methods

SPSS 20.0 statistical software was used for data analysis. Count data were expressed as percentages (%) and compared with the *χ*^2^ test. The rank data were compared by the Wilcoxon rank-sum test. Measurement data of normal distribution were expressed as mean ± SD, and a *t*-test was used for data comparison. *P* < 0.05 indicates that the difference is statistically significant.

## 3. Results

### 3.1. Basic Clinical Information

**Figure 1:**
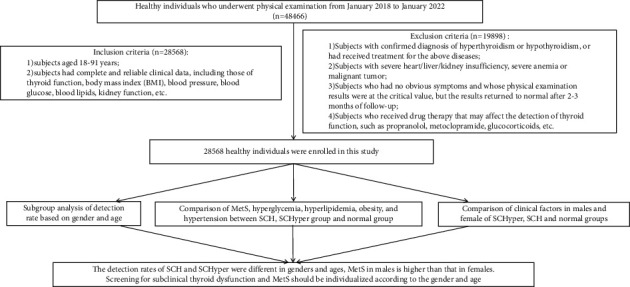
Flow diagram of the analysis process.

A total of 28,568 healthy individuals were included. There were 12,497 males (43.74%) (mean age, 43.51 ± 11.66 years) and 16,071 females (56.26%) (mean age, 39.05 ± 11.23 years). According to the diagnostic criteria of SCH and SCHyper, the study subjects were divided into SCH (*n* = 3170), SCHyper (*n* = 158), and normal groups (*n* = 25240). A detailed flow diagram of the analysis process is presented in Figure 1.

### 3.2. The Detection Rate of SCHyper, SCH, and MetS

Among the 28,568 healthy individuals, subclinical thyroid dysfunction was detected in 3,328 subjects, with a detection rate of 11.64% (95% confidence interval (CI) 11.27-12.03). SCHyper was detected in 158 subjects, with a detection rate of 0.55% (95% CI 0.48–0.63). SCH was detected in 3170 people, with a detection rate of 11.10% (95% CI 10.72–11.47). MetS was detected in 2444 individuals, with a detection rate of 8.56% (95% CI 8.24–8.73). Subgroup analysis was performed based on gender. As shown in Table 1, among the 12,497 males, 47 males had SCHyper, with a detection rate of 0.38% (95% CI 0.29–0.48); 994 males had SCHyper, with a detection rate of 7.95% (95% CI 7.48–8.56); and 1,961 males had MetS, with a detection rate of 15.69% (95% CI 15.03–16.27). Among the 16071 females, 111 individuals had SCHyper (detection rate 0.69%, 95% CI 0.58–0.82), 2176 individuals had SCH (detection rate 13.54%, 95% CI 13.02–14.07), and 483 individuals had MetS (detection rate 3.01%, 95% CI 2.73–3.25). The detection rate of SCHyper and SCH in females was significantly higher than those in males (*P* < 0.001); however, the detection rate of MetS in males was significantly higher than that in females (*P* < 0.001).

### 3.3. Subgroup Analysis of the Detection Rate of SCHyper and SCH Based on Gender and Age

**Figure 2:**
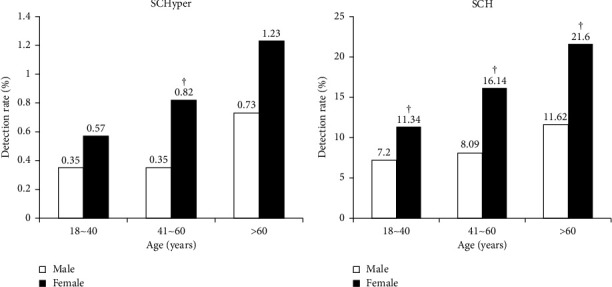
Subgroup analysis of detection rate of SCHyper and SCH based on gender and age. (a) Detection rate of SCHyper. (b) Detection rate of SCH. *Note*. Compared with males of the same age group, ^†^*P* < 0.001.

The subgroup analysis on detection rate of SCHyper based on gender and age showed that the detection rate of SCHyper in females of the age group of 41–60 years was significantly higher than that in males (*P* < 0.001), and there was no statistical significance in other age groups between males and females (*P* > 0.05) (Figure 2(a)). The detection rate of SCHyper was statistically different in different age groups of females (*χ*^2^ = 6.19, *P* = 0.04), while that in different age groups of males was not statistically different (*χ*^2^ = 2.90, *P* = 0.23).

The subgroup analysis on the detection rate of SCH based on gender and age revealed that the detection rate of SCH in females of the same age group was significantly higher than that in males of the same age group (*P* < 0.001) (Figure 2(b)). The detection rates of SCH in all three age groups were significantly different between males and females (in males, *χ*^2^ = 19.34, *P* < 0.001; in females, *χ*^2^ = 109.55, *P* < 0.001).

### 3.4. Comparison of Clinical Factors in Females of SCHyper, SCH, and Normal Groups

**Figure 3:**
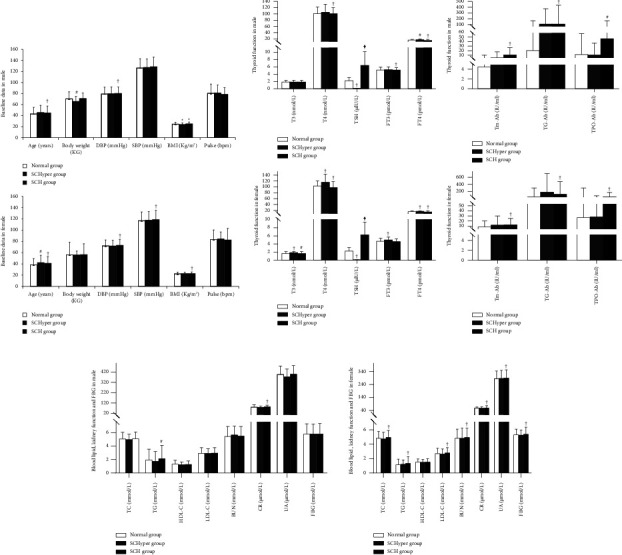
Comparison of clinical factors in males and females of SCHyper, SCH, and normal groups. (a) Baseline data results in male and female. (b) Thyroid function results in male and female. (c) Blood lipid, kidney function, and FBG result in male and female. *Note*. Diastolic blood pressure (DBP), systolic blood pressure (SBP), body mass index (BMI), thyroid peroxidase antibody (TPO-Ab), triiodothyronine (T3), total thyroxine (T4), thyrotropin (TSH), free triiodothyronine (FT3), free thyroxine (FT4), thyroid microsomal antibody (TMTm-Ab), thyroglobulin antibody (TG-Ab), total cholesterol (TC), triglyceride (TG), high-density lipoprotein cholesterol (HDL-C), low-density lipoprotein cholesterol (LDL-C), blood urea nitrogen (BUN), creatinine (CR), uric acid (UA), and fasting blood glucose (FBG). Compared with normal group, ^†^*P* < 0.001, ^#^*P* < 0.01, ^*∗*^*P* < 0.05.

According to the diagnostic criteria of SCH and SCHyper, the females were divided into SCH (*n* = 2176), SCHyper (*n* = 111), and normal groups (*n* = 13680). The factors of age, DBP, SBP, BMI, TPO-Ab, T3, T4, TSH, FT4, Tm-Ab, TG-Ab, TC, TG, LDL, BUN, CR, UA, and FBG in the female SCH group were significantly different from those in the normal group (*P* < 0.01) (Figures 3(a)–3(c)). The female SCHyper group was significantly different from the normal group in the factors of age, T3, T4, TSH, FT3, and FT4 (*P* < 0.01) (Figure 3(b)).

### 3.5. Comparison of Clinical Factors in Males of SCHyper, SCH, and Normal Groups

According to the diagnostic criteria of SCH and SCHyper, the males were divided into SCH (*n* = 994), SCHyper (*n* = 47), and normal groups (*n* = 11560). As shown in Figures 3(a)–3(c), the factors of age, DBP, BMI, TPO-Ab, T4, TSH, FT3, FT4, Tm-Ab, TG-Ab, TG, and CR in the male SCH group were significantly different from those in the normal group (*P* < 0.05). As shown in Figures 3(a) and 3(b), the body weight, BMI, FT4, and TSH of the male SCHyper group were significantly different from those of the normal group (*P* < 0.05).

### 3.6. Comparison of MetS, Hyperglycemia, Hyperlipidemia, Obesity, and Hypertension between SCH Group and Normal Group

**Figure 4:**
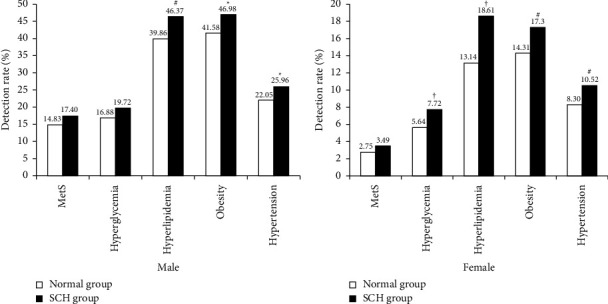
Comparison of MetS, hyperglycemia, hyperlipidemia, obesity, and hypertension between SCH group and normal group. (a) Results in males. (b) Results in females. Compared with normal group, ^†^*P* < 0.001, ^#^*P* < 0.01, ^*∗*^*P* < 0.05.

As shown in Figures 4(a) and 4(b), compared with the normal group, the males in the SCH group were more prone to hyperlipidemia, obesity, and hypertension (*P* < 0.05). In addition, females in the SCH group were more likely to suffer from hyperglycemia, hyperlipidemia, obesity, and hypertension than those in the normal group (*P* < 0.01). However, there was no significant difference in MetS (*P* > 0.05).

### 3.7. Comparison of MetS, Hyperglycemia, Hyperlipidemia, Obesity, and Hypertension between the SCHyper Group and Normal Group

**Figure 5:**
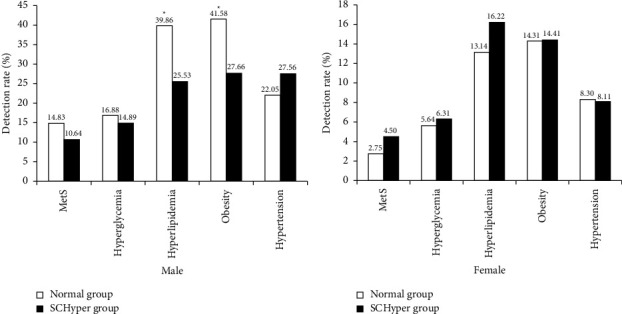
Comparison of MetS, hyperglycemia, hyperlipidemia, obesity, and hypertension between SCHyper group and normal group. (a) Results in males. (b) Results in females. Compared with normal group, ^*∗*^*P* < 0.05.

Compared with SCHyper, the males in the normal group were more likely to suffer from hyperlipidemia and obesity (*P* < 0.05) (Figure 5(a)). However, there was no significant difference between the female SCHyper group and the normal group (*P* > 0.05) (Figure 5(b)). Similarly, no significant difference was found in MetS (Figures 5(a) and 5(b)).

## 4. Discussion

Subclinical thyroid disease is a prevalent clinical issue. Because the majority of individuals are asymptomatic [18], screening becomes crucial for identifying those with the condition. Our large-scale population study revealed the significant role of early screening in detecting subclinical thyroid dysfunction and MetS.

This retrospective analysis found significantly higher detection rates of SCHyper and SCH in females than those in males. The higher detection rates in females may be attributed to the fact that most women in this study were under the age of menopause (58.76%, <40 years, mean age, 31.17 ± 4.89). Previous research has shown a correlation between thyroid hormones and sex hormones, as both the secretion functions of the thyroid and gonads are regulated by the hypothalamic-pituitary axis [19]. Estrogen levels may have an important impact on the mechanism of SCH in females [20]. Therefore, our study highlights the importance of individuals, particularly females, being vigilant about their thyroid function.

The results of the present study demonstrated a relationship between age and the detection rates of SCHyper and SCH. The detection rates were found to increase as age increased. One study reported a prevalence of SCH as high as 10%, particularly among elderly women [7]. In the United States, SCHyper affects 0.7% of the general population [21]. SCHyper is more prevalent among women, older individuals, and those residing in iodine-deficient regions. The frequency of SCHyper increases with age, with rates reaching up to 15.4% in patients aged 75 or older [22, 23]. Both SCHyper and SCH have been associated with coronary heart disease [24]. Despite some discrepancies in screening recommendations, most guidelines support the idea that thyroid function should be assessed in individuals at risk for hypothyroidism, those over the age of 60, and those with known coronary heart disease.

Liu and Chen [25] conducted a study on 58,152 healthy individuals with subclinical hypothyroidism (SCH) in Chongqing, China. They found that the prevalence of SCH in females was 14.76%, which was significantly higher than that in males (7.97%). In this study, the authors retrospectively analyzed the clinical data of 28,568 healthy individuals who underwent physical examinations. Among them, 158 individuals had subclinical hyperthyroidism (SCHyper), and 3,170 individuals had SCH. The overall prevalence of subclinical thyroid dysfunction was 11.64%. The detection rate for males was 7.95%, and for females, it was 13.54%, which was consistent with previous reports [26, 27]. In addition, the detection rate of metabolic syndrome (MetS) in males was about 5 times higher than that in females. This difference may be attributed to factors such as smoking, drinking, and a lack of exercise and dietary control among males.

Dyslipidemia, a common feature of MetS, often manifests in patients with thyroid dysfunction, particularly overt hypothyroidism, leading to increased levels of LDL-C and TC. Limited existing data on TG and HDL-C show varied results in euthyroid subjects [28]. Results for SCH are mostly inconclusive, with some studies [29] demonstrating no difference in HDL-C and TG, while others [30] show elevated values in subclinical forms. In this study, the female SCH group had significantly higher levels of TC, TG, and LDL than the normal group, while the male SCH group had significantly higher TG levels. However, there was no difference in HDL-C levels between the SCH and normal groups for both males and females. Thyroid hormones may disrupt lipid metabolism pathways, particularly in hypothyroidism [31]. Increased TG levels in hypothyroidism are attributed to normal or reduced activity of lipoprotein lipase in adipose tissue and decreased hepatic lipase activity [32]. SCH may affect blood lipids by reducing thyroid hormone levels, leading to increased TC synthesis and decreased conversion of TC to bile acids, vitamin D, and steroid hormones [33]. Furthermore, the coexistence of subclinical thyroid dysfunction with other biochemical abnormalities, such as oxidative stress and insulin resistance, can contribute to dyslipidemia through a vicious cycle [34, 35].

In the present study, we observed that the BMIs of the male and female SCH groups were significantly higher than those of the normal group. Moreover, our study demonstrated that the SCH group was more likely to suffer from hyperlipidemia and obesity in males and females. Thyroid dysfunction is reported to affect BMI [36]. The higher TSH levels could be related to obesity rather than a true hypothyroid state, which could explain the lack of association with MetS in females. Serum TSH values in subclinical thyroid dysfunction, even within the normal ranges, were associated with BMI. Thyroid hormones have been demonstrated to directly induce the expression of 3-hydroxy-3-methylglutaryl coenzyme A (HMG-CoA) reductase in the hepatocyte [37]. Given that the HMG-CoA reductase has been considered a rate-limiting enzyme in LDL-C synthesis [38], suggesting that SCH is associated with an increased risk of hyperlipidemia. Although there is still no conclusion, more and more research results have shown that subclinical thyroid dysfunction is not conducive to the metabolism of blood lipid and blood glucose; that is, SCH has a certain correlation with MetS [39, 40]. Conversely, it was noted that the BMI of the male SCHyper group was lower than that of the normal group, and the normal group was more likely to suffer from hyperlipidemia and obesity than the SCHyper group. Regarding SCHyper states, we have observed a lower occurrence of MetS than expected, considering its association with the relative hypermetabolic state.

In addition, this study found that SCH was closely related to TPO-Ab, Tm-Ab, and TG-Ab. Several studies have shown that the thyroid autoantibodies are indicative of the early symptom of abnormal thyroid function [41–43], suggesting that there may be thyroid autoimmune diseases such as chronic lymphocytic thyroiditis and diffuse goiter with hyperthyroidism (Graves' disease). Screening and intervention of thyroid autoantibodies may help reduce the occurrence and development of thyroid diseases.

In the early stage of abnormal thyroid function, FT3 and FT4 are within the normal range, but TSH, as the most sensitive indicator of thyroid function, may have changed several times or even dozens of times [44]. The heart is the target organ of thyroid hormones. The relationship of subclinical thyroid dysfunction with cardiovascular/cerebrovascular diseases and blood pressure has attracted much attention. This study showed that the SBP and DBP of the female SCH group were significantly higher than those of the normal group, and the DBP of the male SCH group was significantly higher than that of the normal group. Furthermore, it was also observed that the SCH group was more prone to hypertension than the normal group in both males and females. The SCH may regulate blood pressure by impairing ventricular relaxation and filling and affecting vascular smooth muscle cells, resulting in decreased vascular elasticity [45]. However, in this study, the SCHyper was not related to any blood pressure, which may be because FT3 and FT4 are within the normal range and TSH is lower than the normal level. The specific mechanism needs further study.

In this study, it was also observed that subjects in the SCH group had abnormal kidney function. The BUN, CR, and UA in the female SCH group were significantly higher than those in the normal group, and the CR in the male SCH group was also significantly higher than that in the normal group. This may be due to the relative lack of thyroid hormones in the subjects of the SCH group, which may further reduce systemic metabolic levels, and cause the retention of water and sodium between tissues, and the reduction of kidney blood flow [46, 47]. On the other hand, in this study, the FBG was only significantly increased in the female SCH group than in the normal group, but this was not observed in the male SCH group and the male and female SCHyper groups. This may be due to the weak relationship between subclinical thyroid dysfunction and blood glucose.

## 5. Conclusions

In conclusion, attention should be paid to subclinical thyroid dysfunction in elderly people, especially females. Early individualized screening and early intervention should be carried out for individuals with metabolic abnormalities to reduce the damage to target organs caused by subclinical thyroid dysfunction. However, the strength of the current study is a large population-based survey in an iodine-sufficient area. The limitation of this study is a single-centre retrospective analysis. Further prospective studies are needed to confirm the results.

## Figures and Tables

**Table 1:** The detection rate of SCHyper, SCH, and MetS.

Gender	Number of cases	SCHyper		SCH		MetS	
		Number of cases detected	Detection rate (%) (95% CI)	Number of cases detected	Detection rate (%) (95% CI)	Number of cases detected	Detection rate (%) (95% CI)
Male	12497	47	0.38 (0.29–0.48)	994	7.95 (7.48–8.56)	1961	15.69 (15.03–16.27)
Female	16071	111	0.69 (0.58–0.82)	2176	13.54 (13.02–14.07)	483	3.01 (2.73–3.25)
*χ* ^2^		12.65		222.39		1446.31	
*P*		<0.001		<0.001		<0.001	

*Note.* SCHyper, subclinical hyperthyroidism; SCH, subclinical hypothyroidism; MetS, metabolic syndrome.

## Data Availability

The data that support this study will be shared from the corresponding author upon reasonable request.
